# Urolithin A has antiplatelet effects in contrast to its glucuronide, urolithin B, and isourolithin A

**DOI:** 10.3389/fcvm.2026.1843932

**Published:** 2026-09-15

**Authors:** Alina Soloviova, Marcel Hrubša, Carlos J. García, Zuzana Lomozová, Francisco A. Tomás-Barberán, Přemysl Mladěnka

**Affiliations:** 1 Department of Pharmacology and Toxicology, Faculty of Pharmacy, Charles University, Hradec Králové, Czechia; 2 Department of Biotechnology, National University of Pharmacy of the Ministry of Health of Ukraine, Kharkiv, Ukraine; 3 Research Group on Quality, Safety, and Bioactivity of Plant Foods, CEBAS-CSIC, Murcia, Spain; 4 Department of Pharmacognosy and Pharmaceutical Botany, Faculty of Pharmacy, Charles University, Hradec Králové, Czechia

**Keywords:** antiplatelet, coagulation, haemostasis, phenolic, urolithin

## Abstract

**Introduction:**

Consumption of dietary ellagitannins has been suggested to have protective effects against cardiovascular disease, likely due to the colonic formation of urolithins and their subsequent systemic absorption. Urolithins have been previously shown to possess vasodilatory and anti-inflammatory properties. In this study, it was examined whether urolithins (urolithin A, B, and isourolithin A) and two of their glucuronides (urolithin A 3-glucuronide, isourolithin A 3-glucuronide) can block platelet aggregation in humans.

**Methods:**

Seventeen healthy volunteers were included in this study. For the assessment of platelet aggregation, whole blood impedance aggregometry was utilized. Coagulation was examined using a semi-automatic coagulometer with mechanical clot detection. Acute toxicity was evaluated with a rat red blood cell lysis assay. For mechanistic studies, an ELISA-based commercial kit was used.

**Results:**

Urolithin A was a relatively potent antiplatelet drug with an IC_50_ of 3.7 ± 1.0 µM in arachidonic acid-triggered platelet aggregation. Further experiments demonstrated that it could also block platelet aggregation induced by collagen, platelet-activating factor, thrombin receptor activator peptide, adenosine diphosphate, and even dithiothreitol. The findings imply that urolithin A had an effect on the thromboxane A₂ pathway and possibly additional platelet-signalling pathway(s). Combination experiments documented that urolithin can improve the antiplatelet effect of acetylsalicylic acid.

**Conclusion:**

Our observations suggest that urolithin A can be further investigated or used as a template for the development of antiplatelet drug(s). Additional pharmacokinetic, toxicological and efficacy studies are, however, required to confirm and quantify the extent of this effect *in vivo*.

## Graphical Abstract

**Figure:**
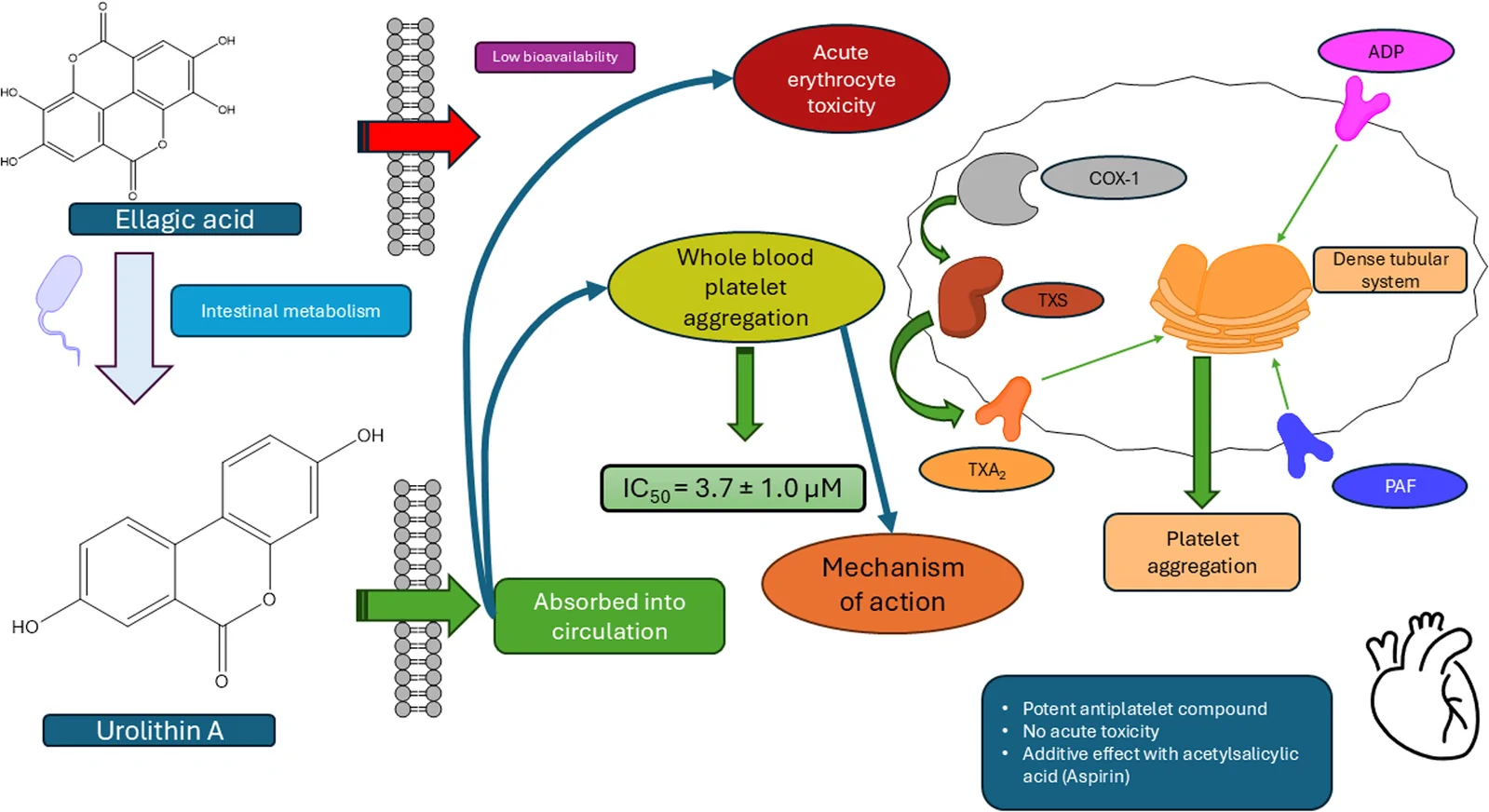

## 1 Introduction

Current antiplatelet treatment suffers from several drawbacks. The gold standard in secondary prevention, acetylsalicylic acid (ASA), is not appropriate for primary prevention of atherothrombotic disease in most cases. Additionally, a degree of low-response, or pseudoresistance, has been observed in a significant number of patients (1, 2). The second-line drugs are the antagonists of purinergic P2Y_12_ receptors. These drugs can be subjected to severe drug interactions and/or also come with an increased risk of severe side effects, mainly bleeding. The last available option, antagonists of the GP_IIb/IIIa_ receptor, are extremely potent and administered parenterally, limiting their application only to healthcare facilities (1, 3). Therefore, a novel drug with a different mechanism of action and/or a better therapeutic profile could improve the outcome of patients with cardiovascular disease, or even provide a feasible option for primary prevention of atherothrombotic disease.

Food phenolic compounds found in most plant-derived products are considered beneficial for cardiovascular health. Due to the immense heterogeneity of structural modifications and the ability of phenolic units to polymerize, many distinct polyphenol groups can be responsible for these effects. Flavonoids, a group of relatively small polyphenols, have shown antiplatelet properties and have been suggested as pivotal food components in maintaining cardiovascular homeostasis (4). They have also been reported as inhibitors of other proteins' aggregation related to neurodegenerative diseases (5). Coumarins, another group of phenolic substances, can possess antiplatelet effects as well (6–8). The mechanisms of these effects are diverse, they depend on the structural features, and this supports their use as important scaffolds for the development of novel antiplatelet drugs (9, 10). Importantly, their activity varies in terms of potency from tens of µM, which is similar to the clinically used drug ASA, to biologically irrelevant concentrations in units of mM (10, 11).

Polymerisation of polyphenolic units can result in ellagitannins, high-molecular weight compounds, which are typically found in pomegranates, black raspberries, raspberries, strawberries, walnuts and almonds. Ellagitannins are also called hydrolysable tannins, as they easily hydrolyse into hexahydroxydiphenic acid that spontaneously lactonizes to form ellagic acid (12). These polyphenols are also of interest, as they exhibit *in vitro* anti-atherogenic, anti-thrombotic, anti-inflammatory, and anti-angiogenic effects at concentrations in a range of 10–100 µM (13). However, the absorption of both ellagitannins and ellagic acid is negligible (14). Hence, to elucidate the systemic impact of these antioxidant phenolics, the focus is given to urolithins, the gut microbiota metabolites produced from the released ellagic acid (15). Ellagitannin-derived urolithins are structurally related to coumarins, as they are benzocoumarins, and therefore could have biological effects on platelet aggregation, although this has not been reported as yet. Urolithin A is currently sold as a dietary supplement both in the US and EU, hailed as a panacea with positive effects on aging, mitochondrial metabolism, brain function and many others. Recent *in vitro* and *in vivo* studies suggest that some beneficial effects are indeed present, but the dosage utilized in these studies, as well as in the dietary supplements, is fairly high (16–18). Data concerning the effect of urolithin A on hemostatic function are currently lacking. As both antiplatelet and anticoagulant drugs are often used in the treatment of cardiovascular disease, this study explores the influence of urolithins on hemostasis. The ability of three urolithins (urolithin A, isourolithin A, urolithin B), as well as their phase II metabolites, to influence hemostasis was evaluated. Their acute toxicity on rat erythrocytes was explored, and additional experiments were designed to suggest a possible mechanism of antiplatelet activity.

## 2 Materials and methods

### 2.1 Chemicals and reagents

Tested compounds: urolithin A (3,8-dihydroxy-urolithin), isourolithin A (3,9-dihydroxy-urolithin), urolithin B (3-hydroxy-urolithin), urolithin A 3-glucuronide (8-hydroxy urolithin 3-glucuronide), and isourolithin A 3-glucuronide (9-hydroxy urolithin 3-glucuronide) with a purity higher than 99%, were provided by VillaPharma Eurofins (Polígono Industrial de Fuente Alamo; Murcia, Spain) (12). Dimethyl sulfoxide (DMSO), platelet activating factor-16 (PAF), acetylsalicylic acid (ASA), 4-methylcatechol, 9,11-dideoxy-9*α*,11*α*-methanoepoxy prostaglandin F_2*α*_ (U-46619), dithiothreitol, calcium ionophore A23187 (calcimycin), bryostatin 1, thapsigargin, terutroban, ticagrelor, cupric sulphate pentahydrate, sodium lactate, potassium phosphate monobasic, ethylenediaminetetraacetic acid disodium salt (EDTA) and Triton X-100 were purchased from Sigma (St. Louis, Missouri, USA). Arachidonic acid (AA), adenosine-5´-diphosphate (ADP), and thrombin receptor agonist peptide-6 (TRAP) were obtained from Roche (Basel, Switzerland), and heparin from Zentiva (Prague, Czech Republic). The potassium phosphate dibasic was sourced from Penta (Chrudim, Czech Republic), while *β*-nicotinamide adenine dinucleotide (NAD+) was from Toronto Research Chemicals (Toronto, Ontario, Canada). The physiological solution was purchased from B.Braun (Melsungen, Germany), whereas collagen from Diagnostica, a.s. (Prague, Czech Republic).

### 2.2 Blood collection

Seventeen recruited blood donors were healthy volunteers aged from 21 to 47 years (median = 26; 11 men/6 women). Informed consent was signed by all healthy volunteers included in this study. Venous blood was collected in heparinized test tubes (17 IU/mL), and the experiment was started within 30 min after blood collection.

### 2.3 Impedance aggregometry

This methodology utilizes human whole blood and various platelet aggregation inducers and inhibitors, which can be used for both evaluating the dose-response relationship and the mechanism of action of tested compounds. This methodology was described thoroughly in our previous publication (19). Briefly, 300 µL of whole blood was diluted with an equal volume of 0.9% sodium chloride preheated to 37 °C and incubated with 5 µL of either tested urolithin, the standard drug, or DMSO (the solvent at a final concentration of 0.8%) for 3 min at 37 °C. Platelet aggregation was then induced with the selected inducer and monitored for 6 min with an impedance aggregometer (Multiplate®, Roche Diagnostic, Basel, Switzerland). Based on our previous studies, two-step calibration using 4-methylcatechol as a standard was employed in the case of AA and collagen. In the case of ADP, TRAP, and PAF, the concentrations used were based on our recent population study (20). In the case of other inducers, a single-step calibration, based on seeking the lowest concentration of the inducer achieving the maximal aggregatory effect, was chosen. The investigators were not blinded during the analysis.

### 2.4 Cyclooxygenase 1 inhibition

The ELISA assessment of cyclooxygenase 1 inhibition was performed with a commercial set from Cayman Chemicals (Ann Arbor, Michigan, United States) according to the manufacturer's instructions (21). Shortly, various concentrations of ASA or urolithin A were incubated with ovine cyclooxygenase 1 at 37 °C, and arachidonic acid, at a final concentration of 100 µM, was added to the mixture to start the reaction. The formed prostaglandin H_2_ was measured following its reduction to prostaglandin F_2*α*_ by stannous chloride. The percentage of inhibition was calculated vs. the positive control containing only the solvent and arachidonic acid.

### 2.5 Combination experiments with clinically and experimentally used antiplatelet drugs

The potential impact of urolithin A on platelet aggregation in the presence of clinically used drugs ASA and ticagrelor, as well as experimental compound terutroban, was assessed in combination experiments. The employed concentrations of these latter drugs were assessed to cause approximately one-third inhibition of platelet activation, allowing for the observation of the potential anti- or proaggregatory impact of urolithin A. The final concentrations were 30 µM for ASA, 100 nM for ticagrelor, and from 49 to 62 nM for terutroban. The experiments were similar to those mentioned above, with the sole exception that the concentration of inducers was fixed: AA was employed at a concentration of 195 μM, U-46619 at 82 nM, and ADP at 5 µM.

### 2.6 Coagulation

Coagulation was determined by measuring coagulation times, i.e., prothrombin time (PT), activated partial thromboplastin time (aPTT), and thrombin time (TT). Normal human control plasma was employed, and the coagulation was measured by the semiautomated coagulometer Ceveron® four (Technoclone, Vienna, Austria). PT was assessed by incubation of 100 µL of plasma with DMSO (final concentration 1%) or DMSO urolithin solution at a final concentration of 50 µM at 37 °C and adding 200 µL of TECHNOPLASTIN-HIS® after a 1-min incubation. aPTT was similar to PT except for the inducer, which was 100 µL DAPTTIN®, followed by 100 µL of 25 mM CaCl_2_. TT experiment was performed using 200 µL of plasma, incubated for 1 min with DMSO or urolithin before the addition of a thrombin reagent. All experiments included heparin as the control (5 IU/mL, 0.5 IU/mL, and 0.1 IU/mL in PT, aPTT, and TT assays, respectively).

### 2.7 Rat red blood cell lysis

This method has been previously reported (22). The erythrocyte suspension was incubated for 4 h at 37 °C with the tested urolithins at various concentrations, which were dissolved in DMSO (final concentration of 1%). DMSO served as a negative control, while cupric sulphate solution in saline (final concentration of 500 μM) was used as a positive control, inducing significant red blood cell lysis. The samples were then centrifuged at 1,950 g for 10 min, and 250 μL of the supernatant was extracted to determine the lactate dehydrogenase (LDH) activity, a marker of cellular lysis. The residual supernatant was removed and replaced with an equal volume of lysis buffer (2 mM EDTA, 1 mM dithiothreitol, 1% Triton X-100, 0.1 M phosphate buffer of pH 7.8). Each sample was vortexed thoroughly and left at room temperature for 20 min to ensure complete lysis of the remaining erythrocytes. The samples were then centrifuged at 1,950 g for 10 min, and 250 μL of the supernatant was used to determine LDH activity. LDH activity was measured kinetically by monitoring the increase in absorbance resulting from the conversion of *β*-NAD⁺ to *β*-NADH. Results were calculated as a percentage of red blood cells lysed. The rat blood used was a by-product from the isolation of the aorta, aimed at testing the vasodilatory effect of compounds, using healthy Wistar Han rats.

### 2.8 Ethics approval

The study in healthy volunteers was conducted according to the guidelines of the Declaration of Helsinki and approved by the Ethics Committee of the Faculty of Pharmacy, Charles University from May 13, 2021 (No. FaFUK/176666/2021-2). Informed consent was signed by all healthy volunteers included in this study. Animal blood was collected from healthy male Wistar Han rats (approval by the Czech Ministry of Health No. MSMT-34121_2017-2). Given the character of our study, no formal statistical power analysis before the initiation of the study was carried out.

### 2.9 Mathematical and statistical evaluation

All mathematical and statistical analysis was performed using GraphPad Prism 11 (GraphPad Software, San Diego, CA, USA). Data are presented as mean ± SD. Normality of data was confirmed using the Shapiro–Wilk normality test. Pairwise comparisons were performed using an unpaired t-test. Multiple data comparisons were analysed by one-way ANOVA followed by the Dunnett´s multiple comparison test. Concentration-response curves were fitted using the least squares regression method. For every data set, measured data were used to create one single fitting curve, and when possible, IC_50_ was calculated from this single curve.

## 3 Results

An initial screening of antiplatelet properties of urolithin A, isourolithin A, their 3-glucuronides, and urolithin B was performed in the first step at a concentration of 80 µM in arachidonic acid-triggered platelet aggregation. Solely, urolithin A was active at this concentration (Figure 1A). Further examination and fitting of the concentration-response curves showed its potent activity (Figure 1B) with a low IC_50_ of 3.7 ± 1.0 µM (95% confidence interval: 2.1–6.4 µM). The IC_50_ of the standard antiplatelet drug, acetylsalicylic acid, is about 10 times higher (31.2 ± 5.8 µM; 95% confidence interval: 22.0–44.0 µM). The other urolithins exhibited antiplatelet activity solely at a high concentration of 160 µM. In the next step, the acute toxicity of urolithins towards erythrocytes was tested to exclude possible biological interference. None of the tested urolithins were toxic to red blood cells up to the maximal tested concentration of 200 µM (Supplementary Figure S1).

**Figure 1:**
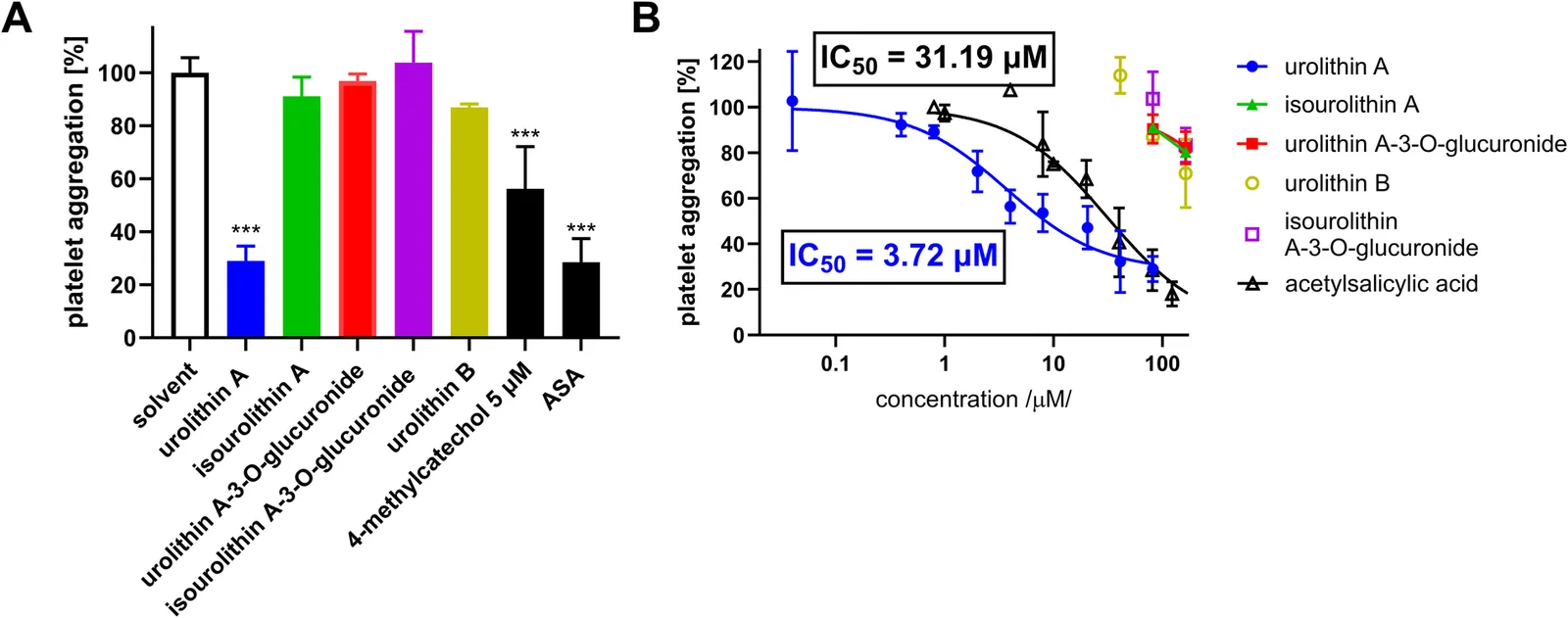
Effect of urolithins on arachidonic acid-based platelet aggregation. **A**: the effect at a concentration of 80 µM (the standard 4-methylcatechol is shown at a concentration of 5 µM), **B**: concentration-dependent inhibitory curves. For construction of the urolithin graphs, blood from four different donors was used. Curves were fitted using the least squares regression method. Statistical comparison in section A was carried out by one-way ANOVA followed by the Dunnett's multiple comparisons test; *** *p* < 0.001 *vs.* the solvent.

Following these results, we concentrated on urolithin A and the mechanism of its antiplatelet effects. Firstly, its effects were confirmed on collagen-based platelet aggregation, as collagen is considered the most biologically relevant trigger of platelet aggregation due to its presence in the subendothelial layer. Also in this case, urolithin A acted as a potent antiplatelet compound (Figure 2).

**Figure 2:**
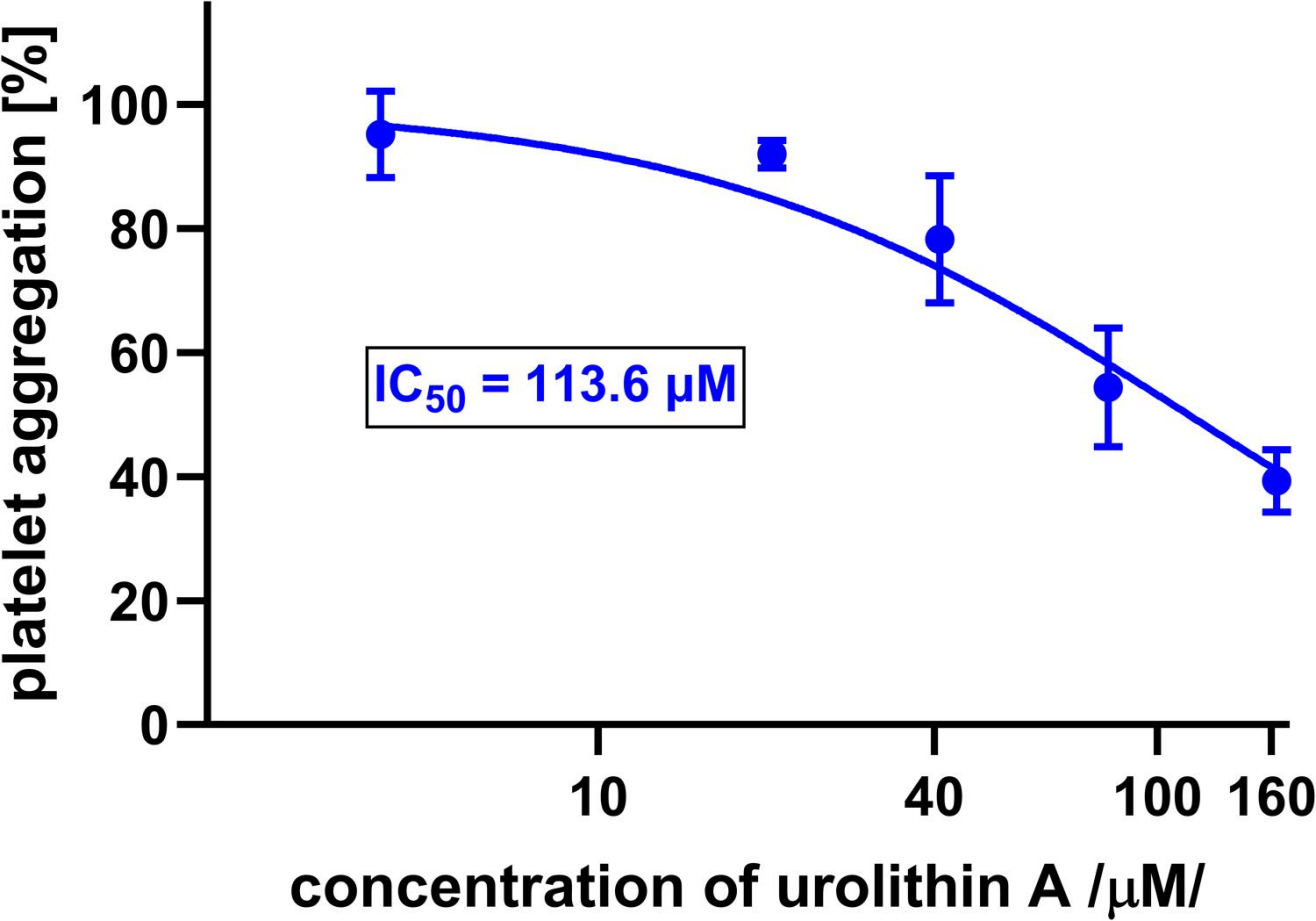
Effect of urolithin A on collagen-triggered platelet aggregation. A dose-response curve was fitted using the least squares regression method. Data are from three different blood donors.

As the effect of urolithin A on arachidonic acid-triggered aggregation was more potent, we have further explored the AA-pathway. Urolithin A did not block cyclooxygenase 1 (Figure 3A), but it was able to diminish platelet aggregation initiated by the terminal platelet metabolite of arachidonic acid, thromboxane A_2_, or precisely by a stable agonist at thromboxane A_2_ receptors, U-46619 (Figure 3B).

**Figure 3:**
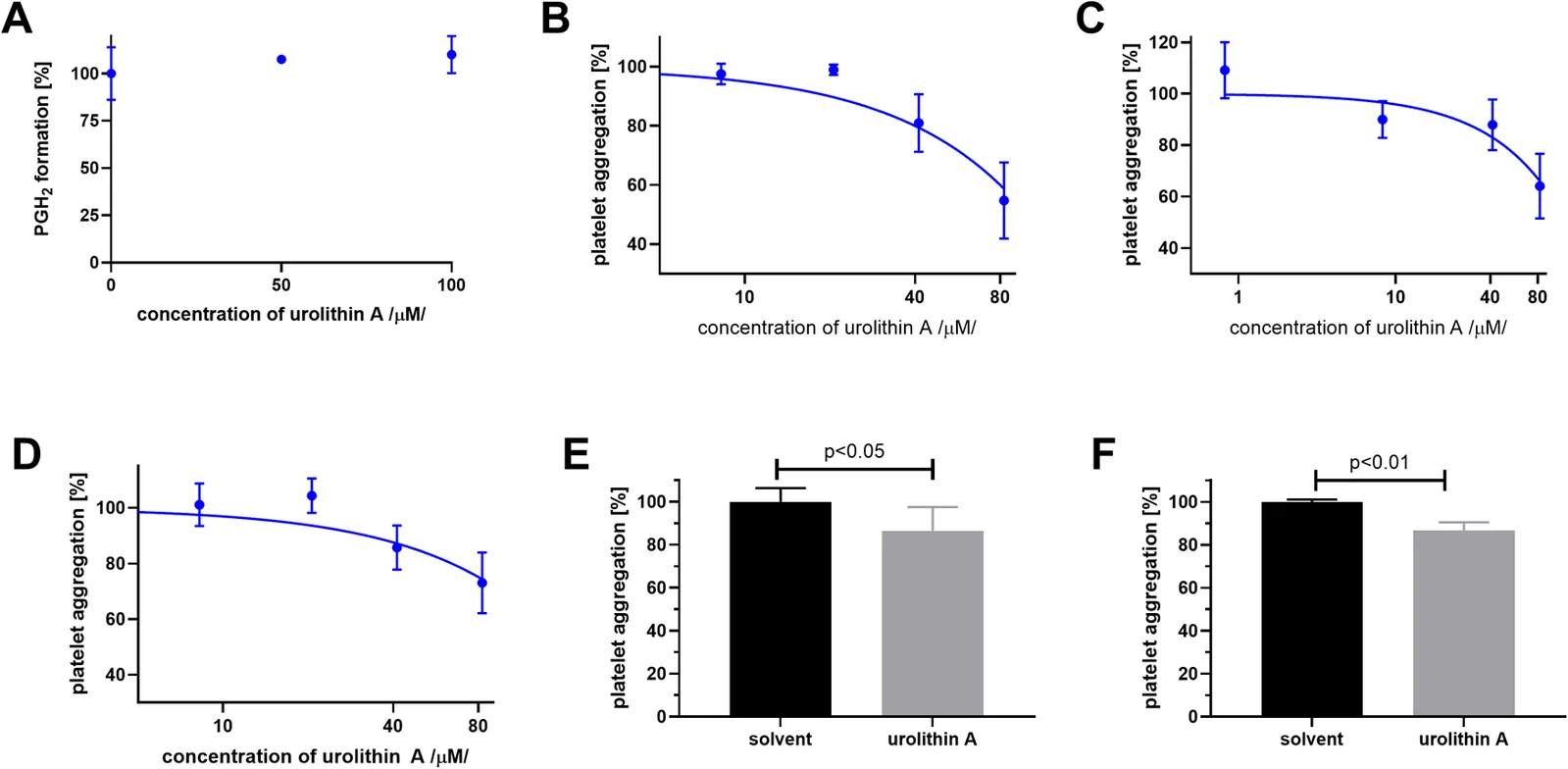
Effect of urolithin A on different platelet aggregation pathways. **A**: no effect of urolithin A on recombinant ovine cyclooxygenase 1, **B**: platelet aggregation triggered by U-46619, **C**: platelet aggregation stimulated by calcimycin, **D**: platelet aggregation triggered by platelet activating factor (PAF), **E**: platelet aggregation initiated by thrombin receptor-activating peptide (TRAP) 16, **F**: platelet aggregation triggered by ADP. In the case of human platelet aggregation **(A–F)**, three independent donors were used for the construction of these graphs. The concentration of urolithin A in graphs **E** and **F** was 80 µM; comparisons in these sections were carried out by an unpaired T-test. Dose-response curves were fitted using the least squares regression method.

Inhibition of the U-46619-mediated platelet aggregation by urolithin A was numerically less potent than the inhibition observed with AA-mediated aggregation. Although we cannot exclude a partial antagonistic effect on thromboxane receptors, the impact of urolithin A on intracellular cascades was probable. Therefore, the focus was moved to intracellular mediators, which may be responsible for this phenomenon. First, the effect of increased intracellular calcium, a central key mediator of platelet aggregation, was investigated. In this assay employing calcimycin, able to transport calcium into the intracellular space, urolithin A again showed to be active (Figure 3C). Based on this effect, the antiplatelet effects against other relevant inducers of platelet aggregation were expected. Indeed, urolithin A blocked platelet aggregation triggered by PAF (Figure 3D), TRAP, as well as ADP (Figures 3E,F). In the latter cases, inhibition was observed solely at the highest concentration of 80 µM. Finally, we have explored the ability of urolithin A to impact the final step of platelet aggregation mediated by the GP_IIb/IIIa_ receptors. Urolithin A was able to partly block platelet aggregation induced by dithiothreitol (Figure 4), which is known to activate GP_IIb/IIIa_ receptors.

**Figure 4:**
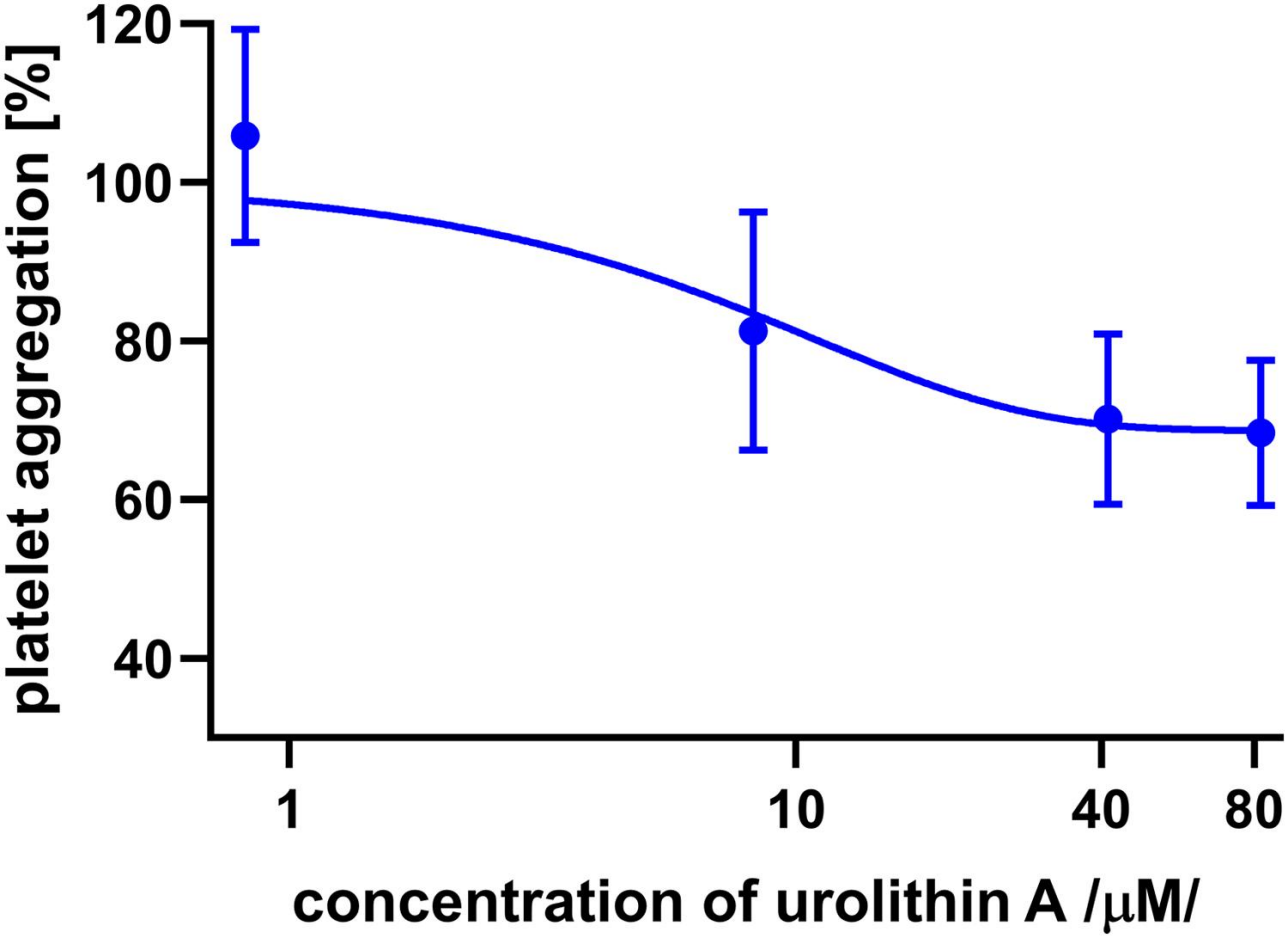
Effect of urolithin A on dithiothreitol-triggered platelet aggregation. A dose-response curve was fitted using the least squares regression method. Data are from four blood donors.

To gain additional mechanistic information as well as assess the impact of urolithin A on the effect of clinically and experimentally used antiplatelet compounds, combination experiments were carried out. Urolithin A was given together with ASA, or a clinically used antagonist at ADP receptors—ticagrelor or an experimentally used thromboxane receptor antagonist, terutroban. In all cases, urolithin increased the effect of the co-administered drugs. The potency of the effect was, however, different. The effect of ASA was improved from a concentration of 20 µM, while that of the other two compounds was increased solely at the highest concentration of 80 µM (Figure 5).

**Figure 5:**
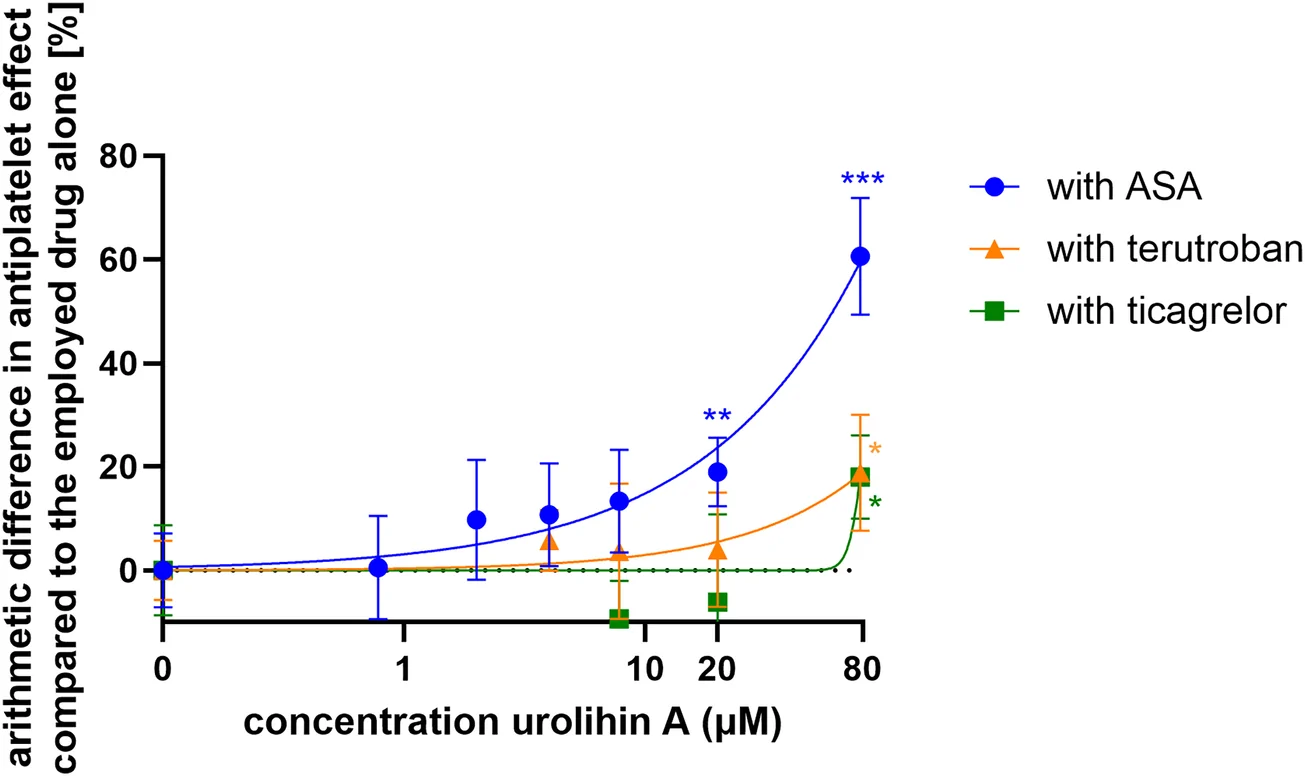
Additive effect of urolithin A on inhibition of platelet aggregation mediated by clinically used drugs (acetylsalicylic acid, ASA) and ticagrelor or experimentally used terutroban. Data are from three independent blood donors. * *p* < 0.05, ** *p* < 0.01, *** *p* < 0.001 vs. the drug alone without urolithin A. One-way ANOVA followed by the Dunnett's multiple comparisons test used to test the differences *vs.* the antiplatelet drug alone (ASA, terutroban or ticagrelor; shown as concentration 0 in the graph). Dose-response curves were fitted using the least squares regression method.

The impact of urolithin A and other investigated urolithins, or their glucuronides on blood coagulation was also tested. None of the compounds at a concentration of 50 µM impacted prothrombin, activated partial thromboplastin, or thrombin times (Supplementary Figure S2).

## 4 Discussion

Polyphenolic compounds have garnered considerable attention in the scientific community due to their purported effects on the cardiovascular system (23–25). Ellagitannins represent one of the groups of polyphenols, which may be responsible for these positive effects (23), and pomegranate, which is a rich source of ellagitannins, has also been associated with the mentioned cardiovascular effects (26). Polyphenols are, however, not at all or only poorly absorbed in their parent forms (27) and hence the observed effects are likely mediated by their metabolites (19, 23, 28). Indeed, ellagitannins are metabolized in the gastrointestinal tract to ellagic acid (29), but this derivative is also poorly absorbed in the small intestine (14). However, studies have confirmed that ellagic acid is metabolized by the gut microbiota in the colon into urolithins. These metabolites are well absorbed (23, 30, 31) and can also be given orally, making them potential drugs (18). There are also specific studies reporting various positive biological effects of urolithins on the cardiovascular system. They include lipid-lowering effects, vasodilatory activity, insulin-sensitizing and other antidiabetic effects, as well as anti-inflammatory effects (13, 18, 32–36). Contrarily, relevant direct antioxidant effects are highly improbable, as they were observed to a very limited extent or were completely lacking (30, 37). In this study, it is reported for the first time that urolithin A has antiplatelet activity, which was approximately 10 times higher than the clinically used drug ASA, at least in our *ex vivo* human platelet model.

Platelets are crucial blood cells primarily responsible for protection against blood loss, but more recent investigations have reported their additional roles in cancer and immune reactions (38). On the other hand, enhanced platelet activation is commonly observed in atherosclerosis, as this pathological unit is associated with endothelial damage (39). Indeed, the uncovering of collagen due to atherosclerotic plaque vulnerability is the first step in pathological thrombus formation, with the subsequent risk of acute myocardial infarction. For this reason, antiplatelet drugs like ASA are used clinically (40). Interestingly, this activation of platelet aggregation by collagen was also, at least partly, blocked by urolithin A in our study (Figure 2). It should be noted that a complete inhibition of this pathway is not suitable, as this will be associated with a higher risk of bleeding, a common side effect of potent antiplatelet drugs.

If we look at the previously reported mechanisms of urolithin A, none of them is apparently relevant for platelets. Urolithin A was shown to have an anti-inflammatory effect due to interference with the NF-*κ*B pathway (32, 41) but not via direct inhibition of cyclooxygenases. The former effect is not relevant in acute experiments in platelets and the absence of the latter effect, i.e., inhibition of cyclooxygenase 1, was also confirmed in this study (Figure 3A). Another mechanism was suggested in a mice model of myocardial ischemia/reperfusion, where activation of phosphoinositide 3-kinase(PI3 K)/Akt pathway was found as a protective mechanism mediated by urolithin A (42). This is not the case in platelets, as the activation of PI3 K/Akt in platelets causes their aggregation (40, 43), and urolithin A had the opposite effect. The mechanism of the vasodilatory activity was not investigated in detail, but this effect, in contrast to the above-mentioned effects, might be potentially similar to the mechanism of antiplatelet activity. On the other hand, a strong vasodilatory effect was observed for both urolithin A and B as they were active at 10 µM or even at lower concentrations (36). In our case, solely urolithin A, but not urolithins B, was an active antiplatelet agent, suggesting the mechanism is not the same. Interestingly, also in the case of another colonic metabolite of food polyphenols, 4-methylcatechol (4-methyl-1,2-dihydroxybenzene), the effect on vessels is mediated by a different mechanism than in platelets (19, 28, 44).

Thromboxane A_2_ causes both vasoconstriction and platelet aggregation. As a vasoconstrictor, it merely serves as one of many mediators. Its involvement in platelet aggregation seems to be essential (45) as it is formed after contact of platelets with subendothelial collagen and shows potent pro-aggregatory properties. Blockade of platelet aggregation initiated by U-46619 might be either carried out by antagonism at thromboxane receptors or mediated by a downstream mechanism. Our data suggest, that antagonism at thromboxane A_2_ receptors was not the primary mechanism of action since the inhibition of arachidonic acid-triggered platelet aggregation (Figure 1A) was stronger than the effect on U-46619-initiated platelet aggregation (Figure 3B). We must, however, also admit, that partial antagonism at the thromboxane receptors cannot be fully excluded based on our limited data. Regardless, we imply that urolithin A can rather either interfere with the production of thromboxane A_2_ or act downstream of thromboxane G_q_-coupled receptor. The lack of an effect on cyclooxygenase 1 would support the downstream hypothesis, but it does not completely invalidate it, as there are other players present. Based on the ability of urolithin A to block platelet aggregation induced by different inducers, including the stimulation of the last step in the aggregation cascade, activation of GP_IIb/IIIa_ receptors, more than one mechanism(s) of action is also likely. Although a direct antagonist at GP_IIb/IIIa_ receptor could explain the ability of urolithin A to interfere with multiple platelet aggregation pathways, we are not of the opinion, that this is the primary or major mechanism of action. First, we do not have experimental proof that urolithin A binds to this receptor, and second, the effect on dithiotheitol-induced platelet aggregation was milder than that triggered by arachidonic acid (Figures 1, 4). An in-depth investigation by employing different experimental approaches is needed to obtain a more detailed mechanistic view. Protein kinases are a possible target, as a previous study showed a partial effect on AMP-activated protein kinase (37). Platelet aggregation is, however, a very complex process, and there are still large gaps in understanding the corresponding signalling pathways (40, 43). Hence, it is very difficult to identify all the mechanism(s) participating in the antiplatelet activity of urolithins.

Importantly, urolithin A was also able to increase the effect of clinically used drugs, in particular ASA. A lower effect was also observed in the case of ticagrelor and terutroban. This seems to agree with our other data. The improvement in the antiplatelet effects of ASA can be explained by different mechanisms of ASA and urolithin A, while, particularly in the case of terutroban, the mechanism might be the same, and hence the additive effect is observed solely in higher concentrations of urolithin A.

The structural aspects deserve a more profound discussion. The platelet-specific effect of urolithin A is interesting in the light of very slight structural differences between our tested compounds. The presence of 3 and 8-hydroxy groups appears to be essential because both the absence of 8-hydroxyl (urolithin B), or conjugation of 3-hydroxyl by a glucuronide, resulted in the disappearance of the antiplatelet effect. Interestingly, also moving the hydroxyl from position 8 to position 9 (isourolithin A) caused the same failure to inhibit platelet aggregation. Based on a relatively high potency of urolithin A and limited structure-relationship data from this study, it would be worth investigating the modifications of its structure since there are several carbons [positions 1,2,4,7, (9) and 10] which could be substituted by different functional moieties in order to possibly improve the antiplatelet effect. The benzocoumarin scaffold, which is also present in urolithins, was already shown to be associated with potent antiplatelet effects in tens of micromolar concentrations and was also more active than ASA (7). Logically, scaffold is important but the selection of suitable substituents is essential.

In addition to the investigation of platelet aggregation, the direct impact on the coagulation system was tested. The complete lack of any direct anticoagulant effect of the tested compounds offers valuable insights. On the other hand, the well-known coumarin derivative warfarin, still being used in anticoagulant therapy, inhibits vitamin K recycling and hence the subsequent synthesis of several coagulation factors. The resulting indirect impact on coagulation cannot be assessed in our acute experiment and must be also checked in further studies.

In addition to possible development of a novel antiplatelet drug, our results can also shed additional light on the biological effects of ellagitannins present in our food. Our experiments were not designed to test this hypothesis, but it is worth commenting. It is interesting to emphasize that urolithin A has both antiplatelet and vasodilatory effects, and this could contribute to the observed positive cardiovascular effects of ellagitannins. These polyphenols are found not only in pomegranate, but also in various nuts and berries (13, 31, 32). The plasma levels of urolithins after consumption of ellagitannin-rich foods are highly variable in the human population, but their total concentration reached up to 18.6 µM (30, 32). This is particularly interesting as urolithin A in µM concentrations will both relax vessels and have antiplatelet activity at the same time. Both mechanisms are considered protective against cardiovascular disease, and in fact, physiological vasodilators and antiplatelet compounds, nitric oxide (NO) and prostacyclin, exhibit these effects. Interestingly, activation of endothelial NO synthase by a mixture of urolithin A and B was found in primary aortic endothelial cells (35), but this is again not applicable for our platelet model. On the other hand, it should also be mentioned that the majority of urolithins are in the form of conjugates, which are inactive from both vascular and platelet points of view. Their deconjugation is possible, and indeed, enterohepatic circulation was suggested (18). Even if it is not probable that unconjugated urolithin A will reach units of µM concentrations after neither consumption of food nor administration of food supplements (17), it should be emphasized that some mild antiplatelet effects of urolithin A were already observed concentrations below 1 µM (Figure 1B). Additional important aspect is that urolithin A is well distributed to different organs (34) and this again suggests its possible deconjugation. Our restricted toxicological data shown that urolithin A is safe in relation to red blood cells, as it did not cause any lysis up to a concentration of 200 µM. It is also probable that due to its mild antiplatelet effects in achievable plasma concentrations, it might be relatively free of risk of bleeding, which is a known side effect of approved antiplatelet drugs. In addition, urolithin A has a relatively long half-life of about 17–22 h and can be orally administered in its pure form (18, 32), which potentially supports the above-suggested new way for the development of novel, semisynthetic antiplatelet compounds. The glucuronidation of the 3-hydroxyl group of urolithin A abolished the antiplatelet effect of this compound, therefore, the rate of deconjugation and the possibility of its uptake into both tissues and blood elements need to be thoroughly investigated. A detailed *in vivo* study of the pharmacokinetics of urolithin A, along with monitoring of its uptake into platelets and other blood cells is necessary to reach a definitive conclusion.

In conclusion, this study has shown that urolithin A, but not urolithin B or isourolithin A, is a relatively potent antiplatelet compound able to inhibit platelet aggregation by different inducers in units of micromolar concentrations at least under *ex vivo* conditions. Our findings suggest that its mechanism of action is compatible with an effect involving the thromboxane A₂ pathway and possibly additional platelet-signalling pathways. At least, according to our aggregation data and red blood cell experiments, as well as available studies from other researchers, it seems to have low toxicity. This must, however, be further confirmed in more targeted *in vivo* studies. We suppose that based on our data and current knowledge, urolithin A is worth subsequent *in vivo* testing as a cardiovascular drug or as a scaffold for developing a suitable derivative. Its antiplatelet effects can also possibly contribute to suggested positive cardiovascular effects of dietary ellagitannins. Although our data are promising, they must be considered preliminary, given limitations of our study.

## 5 Study limitations

Our study has several limitations which have to be reported: 1. A small number of healthy subjects were enrolled, and the antiplatelet effects in patients might be different, 2. Based on the above-reported rapid metabolism to its conjugates and low plasma levels of unchanged urolithin A, the extent of antiplatelet effects of urolithin A *in vivo* in real biological conditions is unknown and should be determined in future experiments, 3. the exploration of the mechanism of action was not thorough enough to definitively identify the precise mechanism of action, 4. Potential toxicity of urolithin A should be excluded by inclusion of different models, including *in vivo* testing.

## Data Availability

The datasets presented in this study can be found in online repositories. The names of the repository/repositories and accession number(s) can be found in the article/Supplementary Material.
