# Supplementary material for: Urolithin A has antiplatelet effects in contrast to its glucuronide, urolithin B, and isourolithin A
Source: Front Cardiovasc Med. 2026 Sep 15;13:1843932. doi: 10.3389/fcvm.2026.1843932 (PMC13619422; doi:10.3389/fcvm.2026.1843932)
Supplement: Supplementary file 1 [file Datasheet1.docx]

Supplementary Material

**Supplementary Figure 1. The effect of urolithins on rat red blood cells.** Data on urolithins A, B, and isourolithin are from 4 independent experiments (every experiment was performed in duplicate), while for their glucuronides from 3 experiments. *** p<0.001 *vs.* the blank sample. The final concentration of cupric ions was 500 µM. One-Way ANOVA was used for the comparison followed by the Dunnett's multiple comparisons test.

**Supplementary Figure S2. The effect of urolithins on blood coagulation**. **A**: activated partial thromboplastin time, **B**: prothrombin time, **C**: thrombin time. None of the urolithins or their glucuronides had a significant effect when compared to the solvent (DMSO). The final concentration of all urolithins was 50 µM. One-Way ANOVA did not find significant differences *vs.* the solvent (DMSO).

Link for raw data set which is available in ZENODO: <https://zenodo.org/records/15752086>
